# Interaction of HE4 and ANXA2 exists in various malignant cells—HE4–ANXA2–MMP2 protein complex promotes cell migration

**DOI:** 10.1186/s12935-019-0864-4

**Published:** 2019-06-13

**Authors:** Jing Wang, Lu Deng, Huiyu Zhuang, Juanjuan Liu, Dawo Liu, Xiao Li, Shan Jin, Liancheng Zhu, Huimin Wang, Bei Lin

**Affiliations:** 1 Department of Obstetrics and Gynaecology, Shengjing Hospital Affiliated To China Medical University, No. 36 Sanhao Street, Heping District, Shenyang, 110004 Liaoning China; 2 Key Laboratory of Maternal-Fetal Medicine of Liaoning Province, Laboratory of Obstetrics and Gynecology of Higher Education of Liaoning Province, No. 7 Mulan Road, Xihu District, Benxi, 117000 Liaoning China; 3 Obstetrics and Gynaecology Hospital of Fudan University, Shanghai, China; 4 Department of Gynecology and Obstetrics, Beijing Chaoyang Hospital, Capital Medical University, No. 8 Workers’ Stadium South Road, Chaoyang District, Beijing, 100020 China; 5 Department of Gynecology, Liaoning Cancer Hospital & Institute China Medical University, No. 44 Xiaoheyan Road, Dadong District, Shenyang, 110000 Liaoning China

**Keywords:** Annexin A2, Human epididymis protein 4, Common phenomenon, MMP2, Protein complex

## Abstract

**Background:**

The interaction between human epididymis protein 4 (HE4) and annexin A2 (Annexin A2) has been found in ovarian cancer. However, it is dimness whether the interaction exists in other malignant tumors.

**Methods:**

Real-time PCR, western blotting and immunocytochemistry were used to detect mRNA and proteins expression. Co-immunoprecipitation and double-labeling immunofluorescence were used to detect the interaction among HE4, ANXA2 and MMP2. MTS assay was used to test cell proliferation. Adhesion test was used to test cell adhesion. Flow cytometry was applied to examine cell cycle. The scratch test and Transwell assay was performed to detect the migration and invasion of various malignant cell lines.

**Results:**

Here we show that the overexpression of HE4 and ANXA2 in various malignant cells is a common phenomenon. HE4 and ANXA2 are co-localized in the cytoplasm and membrane of various tumor cells. ES-2 cells which had both high expression of HE4 and ANXA2 were much stronger in proliferation, adhesion, invasion, and migration than other tumor cells. HE4–ANXA2–MMP2 could form a triple protein complex. HE4 could mediate the expression of MMP2 via ANXA2 to promote cell migration progress.

**Conclusions:**

The interaction of HE4 and ANXA2 exists in various types of cancer cells. HE4 and ANXA2 can promote the proliferation, adhesion, invasion, and migration of cancer cells. HE4–ANXA2–MMP2 form a protein complex and ANXA2 plays the role of “bridge”. They performed together to promote cell migration.

## Background

Human epididymis protein 4 (HE4), also known as WFDC2, was discovered in 1991 and named because of its specific expression in epithelial cells of the epididymis [1]. In 1999, Schummer et al. [2] compared genomic hybridizations and found that HE4 was also highly expressed in ovarian cancer, which raised expectations of an early diagnostic test for this insidious cancer. Subsequently, Hough et al. [3] completed a large-scale analysis of the gene expression profiles of ovarian cell lines and tissues using serial analysis of gene expression (SAGE) technologies, and also found high expression of HE4 in ovarian cancer. Compared with cancer antigen 125 (CA125), the positive rate of HE4 is lower in benign disease, which increase the sensitivity of HE4 to the diagnosis of ovarian cancer. In 2003, HE4 was recognized as a serum marker of ovarian cancer [4], leading to increased studies of HE4 in other cancers. However, althought many studies showed that HE4 was highly expressed in ovarian cancer, its expression in other malignant tumors and normal tissues was also high, albeit to different extents. In 2006, Galgano et al. [5] measured the expression of HE4 in normal and malignant tissues by using oligonucleotide and tissue chips. They found that HE4 was highly expressed in normal trachea, the salivary glands and female reproductive tract. Among tumors, HE4 expression was highest in ovarian cancer. High expression level of HE4 was also observed in pulmonary adenocarcinoma, transitional cell carcinoma, and breast and pancreatic cancer. Our preliminary studies have demonstrated that in addition to ovarian cancer [6], HE4 was also highly expressed in endometrial cancer [7], emphasis that how the specificity of HE4 as a serum marker of ovarian cancer needs to be reassessed.

In protein mass spectrometry, we found a HE4 interacting protein, annexin A2 (ANXA2). HE4/ANXA2 interaction was confirmed by GST pull-down and co immunoprecipitation. HE4/ANXA2 promoted the invasion and migration of ovarian cancer cells. In the past 24 years since the discovery of HE4, There’s been amount of research into the HE4 clinical application, but little is known about its biological functions and mechanisms. Our preliminary studies have shown that the proliferation, invasion, migration, drug resistance and other functions of ovarian cancer cells increased after upregulate of HE4 expression, or after treated with HE4 active protein [8–10]. ANXA2, a member of the annexin family, is a calcium-dependent, membrane phospholipid-binding protein [11] present in several cell types that can regulate membrane transport, signal transduction, differentiation, apoptosis and other biological functions of cells [12]. The expression of ANXA2 has been found in various malignant tumors [13]. ANXA2 is closely related to the occurrence and development of several malignant tumors and plays an important role in angiogenesis, proliferation, apoptosis, adhesion, invasion and migration of tumor cells [14].

According to our preliminary studies, ANXA2 is highly expressed in ovarian [15] and endometrial cancers [16], and HE4 and ANXA2 also show co-localization in endometrial carcinoma. However, so far, there is no research reporting whether this interaction exsists in other malignant tumors. This study examined the expression of HE4 and ANXA2 in malignant tumor cells originated from different tissues. We also determined whether the HE4/ANXA2 interaction was ubiquitous in these cells and whether changes of their expression affected biological behaviors such as proliferation, adhesion, invasion, and migration. Overall, we aime to provide a comprehensive evidence for the expression and biological functions of HE4 and ANXA2 in malignant tumors.

## Methods

### Cell culture and transfection

An ovarian cancer cell line (CaoV-3、ES-2、OVCAR3), endometrial carcinoma cell lines (Ishikawa), lung cancer cell line (A549), cervical cancer cell line (Hela), hepatic carcinoma cell line (HepG2), gastric cancer cell line (HGC-27), colon carcinoma cell lines (SW480, HT29) and human embryo kidney cell line (HEK293) were cultured according to the manufacturer’s protocol. All of the cell lines used in the experiment were purchased from the Shanghai Cell Bank of the Chinese Academy of Sciences. ES-2 was propagated in McCoy’s 5A modified medium with 10% fetal bovine serum; HepG2, HEK293 were propagated in DMEM high glucose medium with 10% fetal bovine serum; other cell lines were cultured in RPMI-1640 medium with 10% fetal bovine serum. All the cell lines were grown at 37 °C in a 5% CO_2_/95% air atmosphere and were revived every 3 to 4 months.

Transfection reagent kits are purchased from invitrogen reagent company. pEGFP-N1-ANXA2 plasmids are purchased from Shanghai GenecChem, and according to the plasmid transfection specifications, this plasmid is transfected into HEK293 cells and stably construct cell line 293-A2. The lentivirus-mediated HE4 shRNA sequence is: 5′-GTCCTGTGTCACTCCCAAT-3′, and the control sequence 5′- TTCTCCGAACGTGTCACGT -3′. The targeting sequences were cloned into GV248 vector (Shanghai GeneChem). The depletion efficiency was evaluated by western blot analysis.

### Real-time PCR

Cells were added with Trizol reagent (1 mL per 10^7^ cells) to extract total RNA. cDNA was synthesized according to the RNA reverse transcription kit instructions (TAKARA, DRR037A). The sequences of HE4 gene primer were 5′-TGCCCCCAGGTGAACATTAAC-3′ for forward primer and 5′-CCATTGCGGCAGCATTTCAT-3′ for reverse primer. The sequences of ANXA2 gene primers were 5′-CTGCTCCAGAACCAACCAG-3′ for forward primer and 5′-TGCGGAAGTCACCAGATGT-3′ for reverse primer. The sequences of GAPDH primers were 5′-ATGGAAATCCCATCACCATCTT-3′ for forward primer and 5′-CGCCCCACTTGATTTTGG-3′ for reverse primer. The Light Cycler PCR system (Roche Diagnostics, Mannheim, Germany) was used for real-time PCR amplification and Ct value detection. The melting curves were analyzed after amplification. PCR reactions of each sample were done in triplicate. Data were analyzed through the comparative threshold cycle (CT) method.

### Immnocytochemistry staining

Cells at exponential phase of growth were digested by 0.25% trypsin and cultured in medium containing 10% FBS to prepare single-cell suspension. Cells were washed twice with cold PBS when growing in a single layer, and fixed with 4% para- formaldehyde for 30 min. The expression of ANXA2 and HE4 on cells were detected according to the SABC kit instructions. The working concentrations of primary antibodies against HE4 and ANXA2 used were 1:50 (Abcam, America, Cat. No. ab200828) and 1:300 (Proteintech, America, Cat. No. 66035-1-Ig), respectively. The primary antibody was replaced by rabbit IgG for negative control.

### Co-immunoprecipitation and western blot

Ice-cold RIPA buffer (1 ml) was added to cells, followed by incubation for 30 min at 4 °C. After centrifugation at 15,000×*g* for 30 min at 4 °C, the supernatant was collected and treated with 10 μl of mouse anti-ANXA2 monoclonal (Proteintech, America, Cat. No. 66035-1-Ig) or goat anti-HE4 polyclonal antibody (Santa Cruz Biotechnology, Inc, America, Cat. No. sc-27570) for 3 h at 4 °C. Then, 20 μl of protein A/G PLUS-Agarose (Santa Cruz Biotechnology, Inc) was added, followed by incubation on a rocker platform overnight at 4 °C. The primary antibody was replaced by mouse or goat IgG (Bioss, China) as negative control. Immunoprecipitates were subsequently subjected to 12% SDS gel electrophoresis and analyzed via western blot using rabbit polyclonal HE4 (Abcam, America, Cat. No. ab109298) and mouse monoclonal ANXA2 antibodies (Proteintech, America, Cat. No. 66035-1-Ig). Proteins were visualized using ECL reagent (Thermo scientific ECL). The experiments were repeated three times.

### Double-labeling immunofluorescence method

Cells in the exponential phase of growth were digested with 0.25% trypsin and cultured in medium containing 10% FBS to prepare single-cell suspension. Cells were washed twice with cold PBS when growing in a single layer, and fixed with 4% para-formaldehyde for 30 min. The cells were simultaneously incubated with primary antibodies against HE4 (Abcam, America, Cat. No. ab200828) and ANXA2 (Proteintech, America, Cat. No. 66035-1-Ig). The primary antibody was replaced by rabbit or mouse IgG for negative controls. The working concentrations of fluorescein isothiocyanate (FITC) and tetraethyl rhodamine isothiocyanate (TRITC) were 1:50. Nuclei were counterstained with DAPI. The empirical procedure was performed according to the manufacturer’s instructions.

### Wound healing

Cells during the log phase were selected and seeded into 6-well plates. When cell confluence was 90%, the cells were starved with serum-free medium overnight. Then scrathed the plate straightly with 10 µl pipette. Cells were cultured in medium without serum. After 24 h, the width of the scarification were observed via inverted microscope (DMI3000B, Leica, Germany) with 100× magnification. Wound-healing percentage of the cells was determined by the ratio of healing width at each time point to the wound width at 0 h.

### Invasion assays

Cell invasion assay were performed using transwell chamber with pore size of 8 μm. For the invasion assay, 2 × 10^5^ cells were seeded in 200 μl serum-free medium in the upper chamber coated with matrigel (BD Biosciences, NJ, USA). The pipette tip was pre-cooled, and the ECM gel was melted at 4 °C overnight, diluted by 1:8 with serum free medium. Complete medium was added to the lower chamber. After 24 h or 48 h incubation at 37 °C, cells in the upper chamber were carefully removed with a cotton swab and the cells traversed to the back membrane of the chamber were fixed in 4% para-formaldehyde and stained with 0.1% crystal violet. For quantification, five fields (upper, lower, left, right, middle × 400) per filter were counted under a microscope (DMI3000B, Leica, Germany).

### Cell proliferation test

Cellular proliferation was analyzed by CellTiter 96^R^ AQ_ueous_ One Solution Cell Proliferation Assay (Promega, USA). Cells were seeded at a density of 10^3^ per well in 96-well culture plate. Cell proliferation was detected at 24, 48, 72 h, respectively. 100 μl medium were added with 20 μl MTS, followed by incubation for 2 h. The optical density at 490 nm was measured on an ELX-800 spectrometer reader (Bio-Tek Instruments, Winooski, USA).

### Cell-cycle analysis

Cells were harvested and washed with PBS for three times. Then, they were fixed with 70% cold ethanol at 4 °C overnight. Cells were resuspended and washed with PBS, then resuspended in 100 μl RNase, 37 °C for 30 min. Then added 400 μl propidium iodide (PI), 4 °C in dark for 30 min, and quantified by FACSCalibur (BD Biosciences, San Jose, CA, USA). The empirical procedure was performed according to the manufacturer’s instructions and repeated for three times.

### Adhesion test

Cells were seeded in 96-well plates at a density of 2 × 10^3^ per well and incubated at 37 °C for 10, 30, 60 min, then washed with PBS to remove non-adherent cells. Adherent cells were fixed with 4% para-formaldehyde, after washed by PBS, cells were stained with crystal violet. Washed with PBS and added 2% SDS to terminate the reaction. The optical density at 570 nm was measured on an ELX-800 spectrometer reader (Bio-Tek Instruments, Winooski, USA).

### Statistical analysis

SPSS version 17.0 (SPSS Inc, Chicago, IL) software was used for statistical analysis. χ^2^ analysis, variance analysis, and *t* test were employed. *p* < 0.05 was considered statistically significant.

## Results

### HE4 and ANXA2 were highly expressed in various malignant cells

**Fig. 1:**
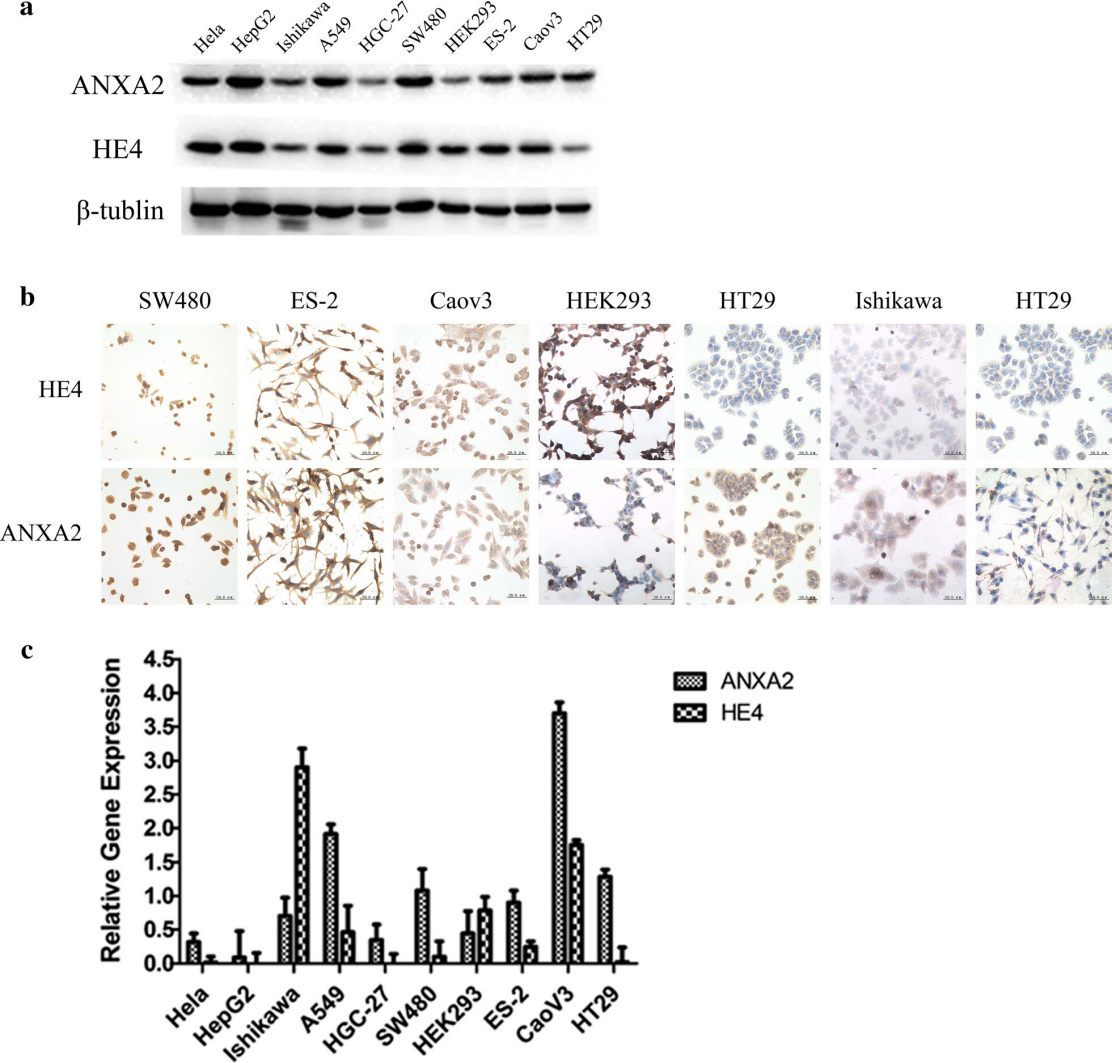
Expression level of HE4 and ANXA2 in various cells. **a** Western blot detected the expression of HE4 and ANXA2 in different cells; **b** expression of HE4 and ANXA2 detected by immunocytochemistry. The scale ruler represents 50.0 μm; **c** gene expression level of HE4 and ANXA2 in different cells detected by real-time PCR

We detected protein and mRNA expression levels of HE4 and ANXA2 in nine types of malignant cells lines (ES-2, CaoV3, A549, HeLa, HepG2, Ishikawa, HGC-27, SW480 and HT29) and Human Embryonic Kidney 293 cell line by western blot (Fig. 1a), immunocytochemistry (Fig. 1b) and real-time PCR (Fig. 1c). The results show that HE4 and ANXA2 were highly expressed in various types of cells including Hela, HepG2, Ishikawa, A549, HGC-27, SW480, HEK293, ES-2, CaoV3, HT29, and their expression in cells was mainly concentrated in the cell membrane and cytoplasm. The results of real-time PCR are not completely consistent with it of western blot and immunocytochemistry, which is because that protein expression levels are not only regulated by the level of genes, but also by the post-translational control.

### Interaction and co-localization of HE4 and ANXA2 are common in various malignant cells

**Fig. 2:**
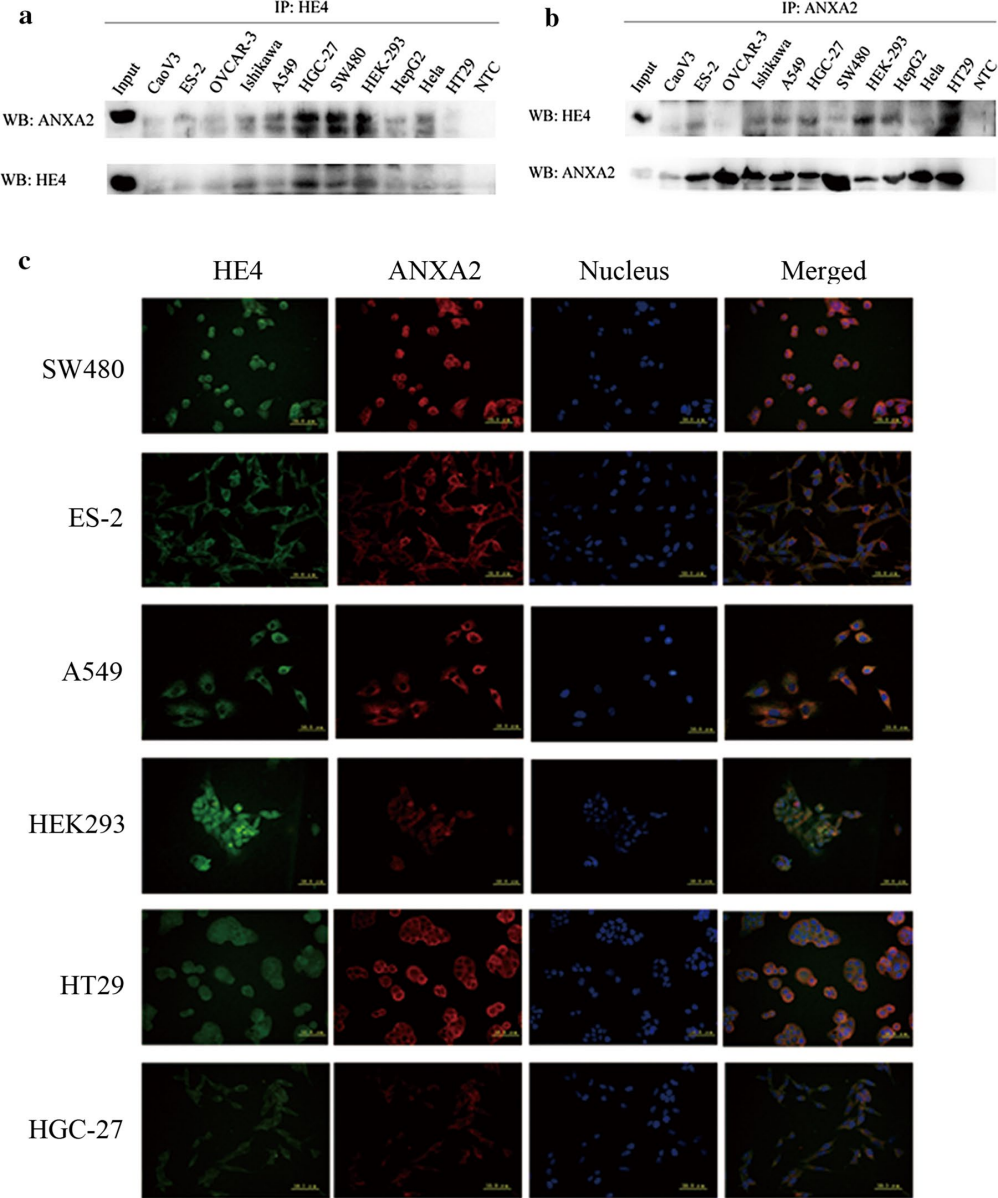
Co-localization of HE4 and ANXA2 in different kinds of cells. **a** Immunoprecipitation of ANXA2-HE4 complex by anti-HE4 antibody and western blot analysis with anti-ANXA2 antibody; “input” is total cell lysates, “NTC” is negative control without protein; **b** immunoprecipitation of ANXA2-HE4 complex by anti-ANXA2 antibody and western blot analysis with anti-HE4 antibody; **c** double-labeling immunofluoscence showed the co-localization of HE4 and ANXA2 in different cells, “blue” represents nucleus; “orange” represents the co-localization of ANXA2 and HE4. The scale ruler represents 50.0 μm

We further studied the HE4-ANXA2 interaction in different cell lines by co-immunoprecipitation and double-label immunofluorescence assays. Co-immunoprecipitation (Fig. 2a, b) revealed that the interaction between HE4 and ANXA2 was in various malignant cell lines including CaoV3, ES-2, OVCAR-3, Ishikawa, A549, HGC-27, SW480, HepG2, Hela, HT29 and HEK293 cell line. The results of the double-label immunofluorescence assay (Fig. 2c) showed that the co-localization of HE4/ANXA2 was on the cytoplasm and membrane of HEK293 and various malignant tumor cells including SW480, ES-2, A549, HT29 and HGC-27.

### Cells expressing higher level of HE4 and ANXA2 show stronger ability of proliferation, adhesion, invasion and metastasis

**Fig. 3:**
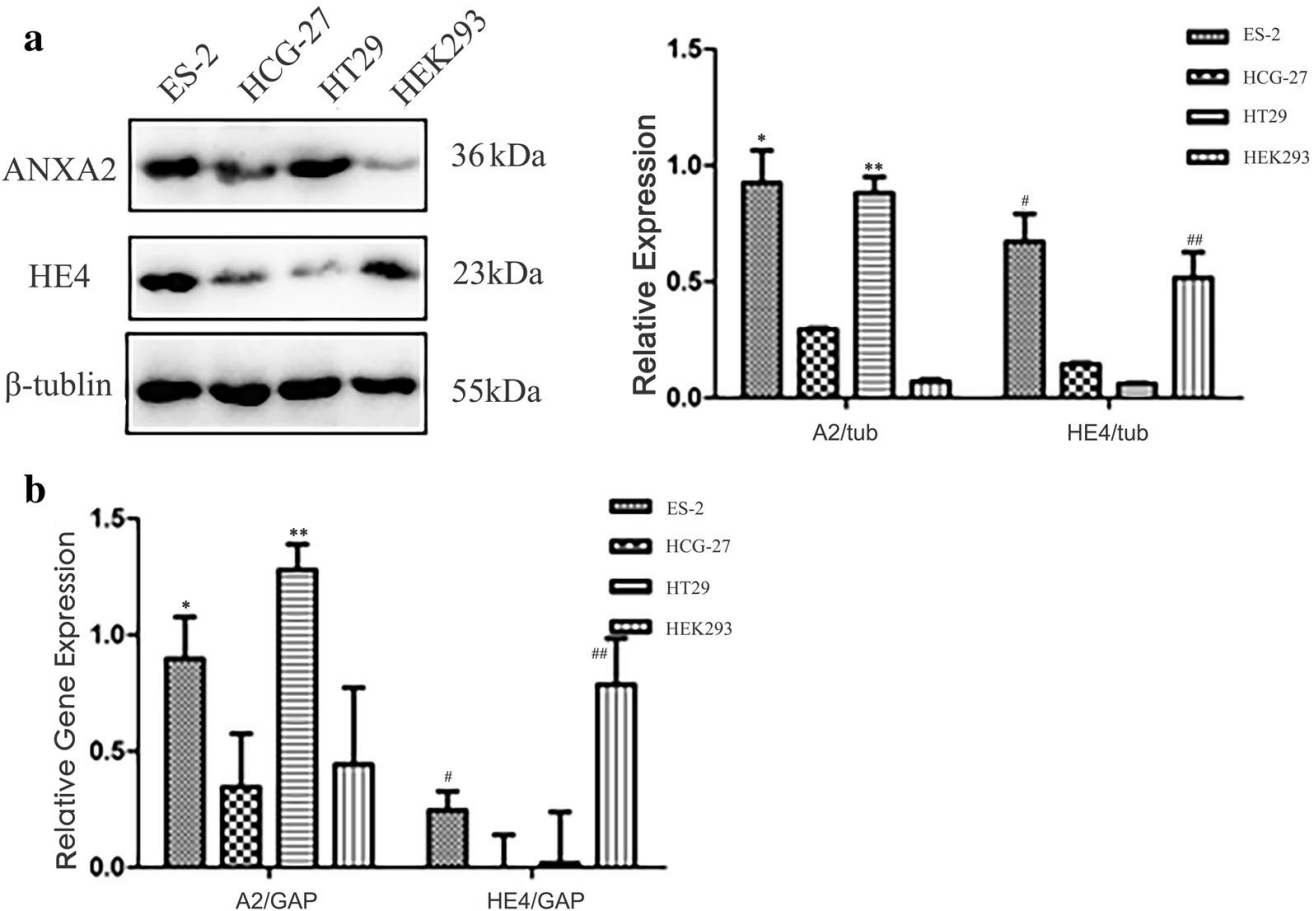
Expression of HE4 and ANXA2 in different cell lines. **a** Expression of HE4 and ANXA2 were detected by western blot; **b** Expression of HE4 and ANXA2 were detected by real-time PCR. * and ** mean expression of ANXA2 in ES-2 and HT29 are significantly higher than HGC-27 and HEK293 cells, # and ## mean expression of HE4 in ES-2 and HEK293 are significantly higher than HGC-27 and HT29 cells, *p *< 0.05

We found several cell lines with distinct expressions of HE4 and ANXA2 gene and protein through western blot (Fig. 3a) and real-time PCR (Fig. 3b): ES-2 has high expression of both HE4 and ANXA2; HEK293 has high expression of HE4 but relatively low expression of ANXA2; HT29 has high expression of ANXA2 but relatively low expression of HE4; HGC-27, with relatively low expressed of both HE4 and ANXA2.

**Fig. 4:**
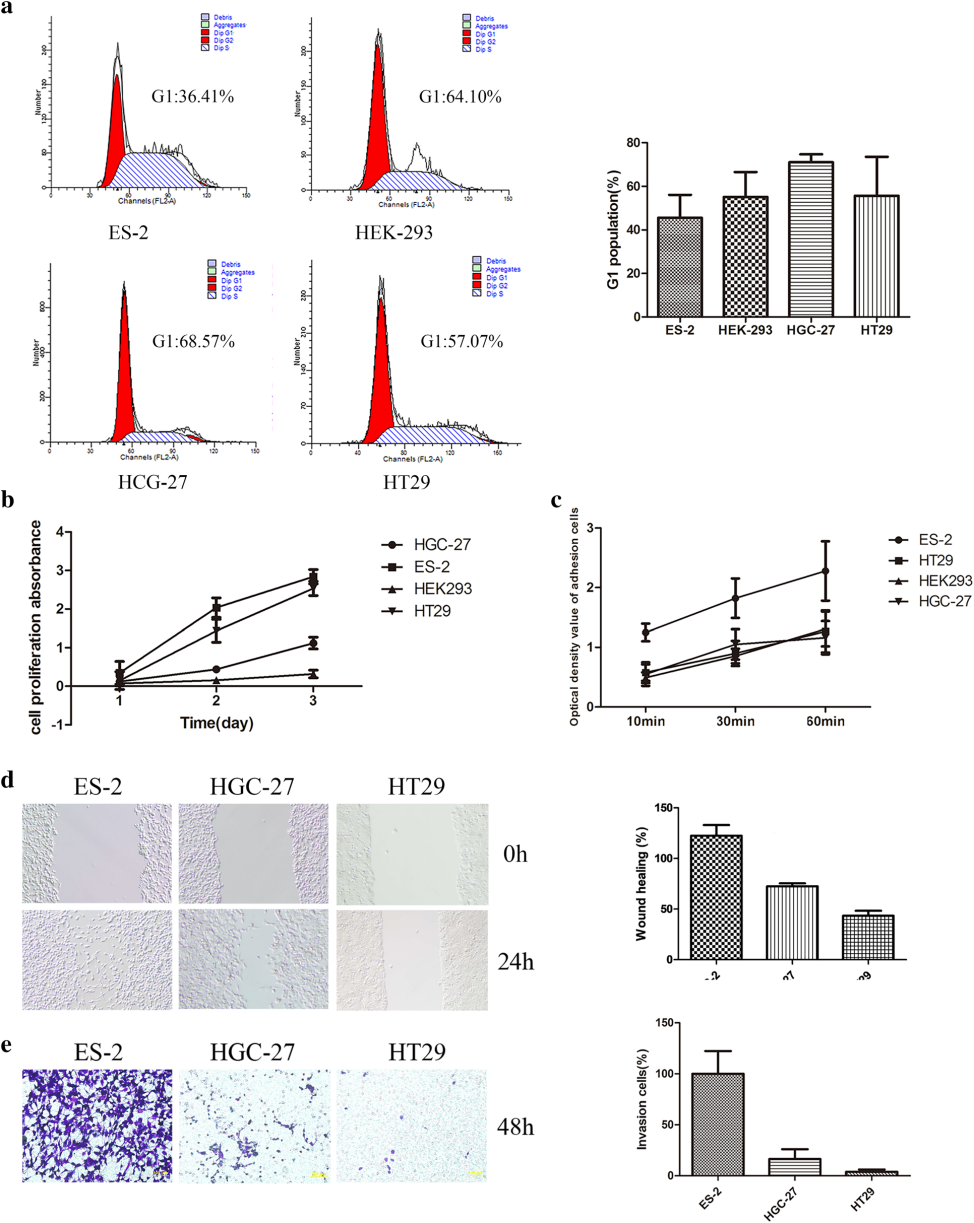
Comparing the biological behaviors in cells with different expression levels of HE4 and ANXA2. **a** Cell cycles are detected by flow cytometry. * means the proportion of G1 phase of ES-2 is significantly less than that of HGC-27 (*p *< 0.05); **b** proliferation ability of different cells are detected by MTS. *and & mean the proliferation abilities of ES-2 and HT29 are significantly stronger than that of HGC-27 and HEK293 (*p *< 0.05); **c** adhesion ability of different cells are detected by adhesion test. * means the adhesion ability of ES-2 is significantly stronger than that of the others (*p *< 0.05); **d** migration ability of different cancer cells are detected by wound healing assay. * means the wound healing ability of ES-2 is stronger than that of HGC-27 and HT29 cells (*p *< 0.05); **e** invasion ability of different cancer cells are detected by matrigel-transwell assay. # means the invasion ability of ES-2 is stronger than that of HGC-27 and HT29 cells (*p *< 0.05)

The cell cycle of above four types of cell lines was detected by flow cytometry. The results showed that the G1 phase of both ES-2 and HT29 cells was lower than that of HEK293 and HGC-27, And the difference was statistically significant (*p *< 0.05) (Fig. 4a). The proliferative capacity of ES-2 and HT29 was significantly higher than that of HGC-27 and HEK293 cell lines by MTS assay (all *p *< 0.05). Both ES-2 and HT29 cell lines are highly expressed of ANXA2, which indicated that ANXA2 may play an important role in the proliferation of malignant cells (Fig. 4b).

Cell adhesion plays an important role in drug resistance, invasion and metastasis of malignant tumors. We compared the adhesion capacity of above four types of cells by adhesion experiments. The results showed that the number of ES-2 cells adhering to the culture plate at 10 min, 30 min, and 60 min was significantly higher than that of the other three type of cell lines (*p *< 0.05). ES-2 has high expression of both HE4 and ANXA2, which indicated that a concomitant high expression of HE4 and ANXA2 might promote the adhesion behavior of tumor cells (Fig. 4c).

The migration ability and invasive ability of the cells were detected by scratch test, matrigel-transwell chamber assay. The results showed that migration and invasion ability of ES-2 was significantly stronger than those of HGC-27 and HT29 (*p *< 0.05). HE4 expression level of ES-2 was higher than that of HGC-27 and HT29, which both cell lines had relatively low expressing of HE4. The above results suggested that HE4 might play an important role in invasion and metastasis of tumor cells (Fig. 4d, e).

### HE4–ANXA2–MMP2 form a protein complex which works together to promote cell migration

**Fig. 5:**
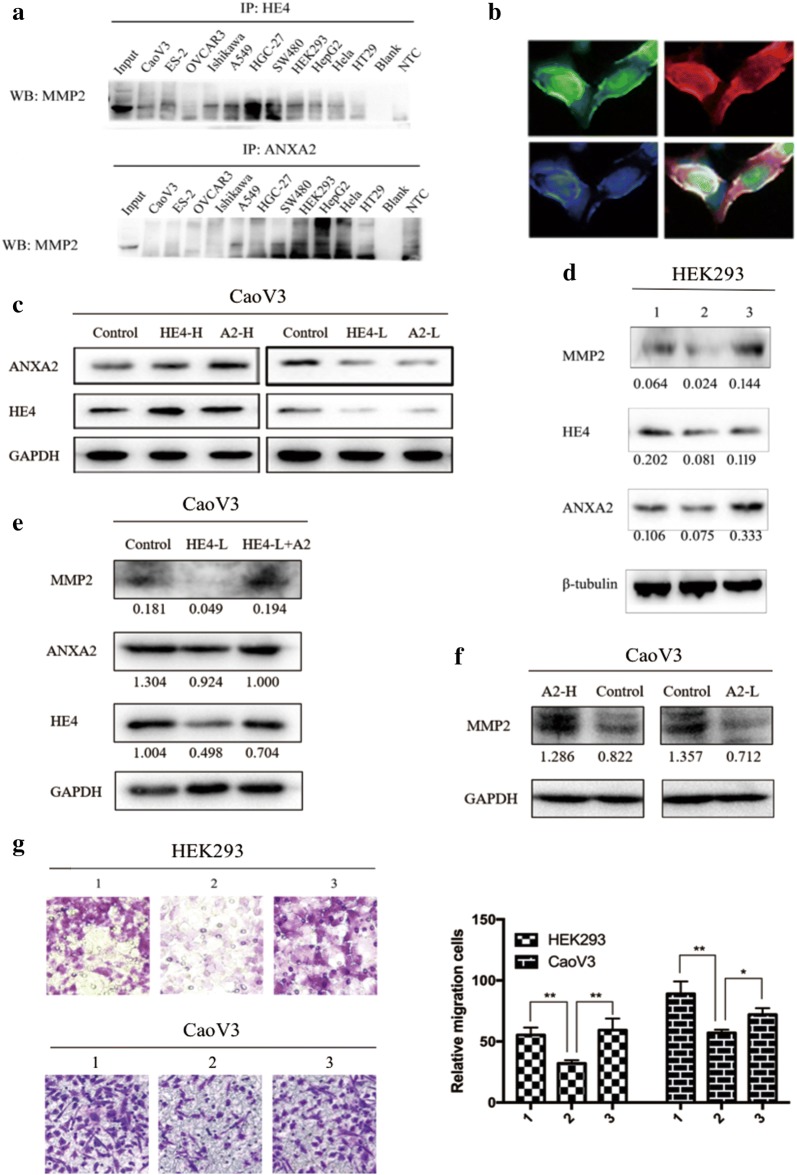
HE4–ANXA2–MMP2 protein complex work together to promote cell migration. **a** Immunoprecipitation of HE4 or ANXA2, and western blot detection of MMP2; **b** immunofluorescence detection of co-localization of ANXA2 and MMP2 in HEK293 cell. Green represents MMP2, red represents ANXA2, blue represents nucleus, and orange is the merged color of green and red; **c** western blot detection of Caov-3 cell model which was stably transfected with HE4 or ANXA2 gene or shRNA; **d**, **e** western blot detection and **g** migration detection after transfection and protein addition. 1 is control cells, 2 is HE4-silencing cells, 3 is HE4-silencing with recombinant ANXA2 addition. **f** Western blot detection of MMP2 with the change of ANXA2 in transfected Caov-3

Co-immunoprecipitation was used to detecte the interaction between HE4/ANXA2 and MMP2. The results showed interaction between HE4/ANXA2 and MMP2 was common in various malignant tumor cells including CaoV3, ES-2, OVCAR-3, Ishikawa, A549, HGC-27, SW480, HepG2, Hela, HT29 and HEK293 cell lines (Fig. 5a). Immunofluorescence assay revealed co-localization of ANXA2 and MMP2 in HEK293 cells and they were co-expressed in cell membranes and cytoplasm (Fig. 5b).

The result of western blot and transwell migration experiments demonstrated the expression of ANXA2, MMP2 and ability of cell migration decreased when HE4 gene was silenced in HEK293 and CaoV3 cell lines, and recovered after adding human recombinant ANXA2 active protein (Fig. 5c–e, g). At the same time, ANXA2 over or down-expression caused the same changes in MMP2 expression (Fig. 5f). The above results suggested that HE4 might regulate MMP2 expression via ANXA2 to affect cell invasion and migration.

## Discussion

HE4 protein is encoded by the *WFDC2* gene and contains two WAP domains; it is considered to be homologous to the extracellular proteinase inhibitor, SLPI protein because of its structural specificity. HE4 has been found in the epithelial cells of the distal spermatic duct and may be involved in sperm maturation [1]. Subsequent studies [2] showed that HE4 was very highly expressed in ovarian cancer but barely expressed in normal ovarian epithelium, which made it an ideal candidate marker for ovarian cancer with approval for its use in a clinical application [4]. However, additional studies demonstrated that, in addition to ovarian cancer, HE4 expression was not only in normal tissues but also in both benign and malignant diseases to varying degrees [17], which has serious doubts about the specificity of HE4 and its use as a biomarker for this cancer [18]. The biological function of HE4 in malignant tumors is also controversial. Some investigators suggested HE4 played an inhibitory role in the proliferation and migration of malignant tumors or certain benign lesions. A study completed by Gao et al. [19] showed that HE4 facilitated the apoptosis and adhesion of ovarian cancer cells, while inhibiting the proliferation, migration, and invasion of these cells. In 2013, Kong et al. [20] found, using in vitro stable transfection techniques, HE4 could suppress the proliferation of ovarian cancer cells. These authors showed that HE4 inhibited the proliferation of ovarian cancer cells via MAPK and PI3K/AKT signaling pathways in vitro, but did not affect EGFR phosphorylation. Other investigators proposed that HE4 promoted the proliferation, drug resistance, invasion, and migration of ovarian or endometrial cancers. In 2012, Lu et al. [21] found, in in vivo and in vitro studies, that HE4 regulated EGFR and Erk1/2 phosphorylation to promote the proliferation of ovarian cancer. In 2013, Moore et al. [22] further found that a high HE4 serum level was related to drug resistance and a shortened survival time for patients with epithelial ovarian cancer. This group also demonstrated that a increased HE4 expression facilitated the growth of tumor and chemotherapy resistance to cisplatin, leading to decreased survival rate in nude mice. However, a decreased HE4 expression inhibited tumor formation in nude mice and HE4 could respond to tumor microenvironment components. EGFR, IGF1R and HIF1α interacted with HE4, the expression of which increased and nuclear translocation occurred after EGF, VEGF and insulin treatment. These results suggested that HE4 probably played an important role in the formation of ovarian cancer tumors. In 2011, Moore et al. [23] found, that serum HE4 expression was associated with muscular invasion of endometrial cancer. Furthermore, a study in 2013 by Li et al. [24] demonstrated that HE4 promoted proliferation and invasion of an endometrial cancer cell line. A study completed by Lu et al. in 2015 [25] showed that treatment with human HE4 recombinant active protein increased the viability and proliferation of pancreatic and endometrial cancer cells, and that cell lines with highly expressed levels of HE4 caused subcutaneous tumor formation in nude mice.

In response to the controversies surrounding the biological functions of HE4 in ovarian cancer cells, our research group previously examined these biological functions in in vivo and in vitro models by transfection and silencing of HE4, and by adding HE4 active protein to cultured cells [8, 9, 26, 27]. We found that HE4 promoted not only proliferation, but also the invasion and migration of ovarian cancer cells. Additionally, HE4 expression in ovarian cancer tissues positively correlated with the adhesion molecules CD44, integrin β1, and integrin α5.

ANXA2 is a HE4-interacting protein in ovarian cancer which was discovered by our research group through mass spectrum analysis in the preliminary phase [9]. ANXA2, a member of the annexin family, is a calcium-dependent membrane phospholipid-binding protein that plays an important role in several physiological and pathological processes, particularly in tumorigenesis [15]. ANXA2 is usually present in a monomer or heterotetramer form. The ANXA2 monomer exists in the cytoplasm, while the ANXA2 heterotetramer is located in the membrane and is composed of two ANXA2 monomers and two p11 (S100A10) protein monomers [28]. The ANXA2 monomer translocates to the cell membrane due to heat stress, tyrosine phosphorylation, and by interaction with heat shock protein 90α [29–32]. Intracellular ANXA2 is involved in such physiological processes as exocytosis, endocytosis and membrane transport [33–36]; its interaction with CD44 plays an important role in lipid raft formation and signal transduction [37]. ANXA2, as a radioreactive protein, can hinder radiation-induced apoptosis by regulating NF-κB nuclear translocation [38].

In order to further substantiate whether HE4 and ANXA2 interaction is ubiquitous in malignant tumor cells, we detected the expression and interaction of HE4 and ANXA2 in different malignant tumor cell lines, and then compared the differences between the proliferation, invasion, migration and adhesion of cell lines with different expressions level of HE4 and ANXA2. The results showed that HE4 and ANXA2, both located in the membrane and cytoplasm, were highly expressed and widely interacted in various malignant tumor cells. Cell lines that expressed high levels of HE4 and ANXA2 showed the greatest ability in invasion, migration, proliferation, and adhesion. Cell lines with high level of ANXA2 showed higher proliferation ability.

MMP2, is a matrix hydrolase that specifically dissolves IV collagen in matrix metalloproteinase family; Its expression is positively correlated with tumor progression, metastasis, and poor prognosis [39, 40]. A study by LeBleu et al. [41] showed that HE4 expression in fibrotic renal tissues was high, and the activities of MMP2 and MMP9 were suppressed. What is the mechanism by which HE4 promotes invasion and metastasis in malignant tumor cells? Studies have shown that extracellular ANXA2 acted as a co-receptor for tissue plasminogen activator and plasminogen to promote fibrinolytic protein formation, activate MMPs and promote degradation of extracellular matrix which provided favorable conditions for cell to perform invasion and metastasis [42]. In pulmonary adenocarcinoma and liver cancers, decreased expression of ANXA2 after ANXA2 knock-down was followed by decreased expression of MMP2, as well as decreased proliferation, migration and invasion ability [43, 44]. We examined the interaction among HE4, ANXA2 and MMP2 in various malignant tumor cell lines and the tool cell line HEK293, according the results, it was inferred that HE4, ANXA2, and MMP2 might form a protein complex. We constructed HE4 -silenced HEK293 cell line and HE4 or ANXA2 over- or down-expression CaoV3 cell line model, found that the expression of ANXA2, MMP2 and ability of cell migration decreased when HE4 gene was silenced, and recovered after adding human recombinant ANXA2 active protein. At the same time, ANXA2 over or down-expression caused the same changes in MMP2 expression. Therefore, we speculated that HE4 might affect the expression and function of MMP2 via ANXA2, thereby promoting cell metastasis.

In conclusion, this study has revealed that HE4 is expressed in several malignant tumor types where it interacts with ANXA2 to facilitate invasion, migration, adhesion, and proliferation. HE4, ANXA2 and MMP2 can form a protein complex. HE4 also promotes cellular invasion and migration through influencing MMP2 expression via ANXA2. These findings provides an important experimental basis for the biological function of HE4 and ANXA2 in the progression of malignant tumors.

## Conclusions

In summary, the interaction of HE4 and ANXA2 commonly exists in various cancer cells. HE4 and ANXA2 can synergistic promote the proliferation, adhesion, invasion, and migration of cancer cells. In addition, HE4 and MMP2 form a protein complex and ANXA2 plays the role of “bridge”. They work together to promote cell migration.

## Abbreviations

HE4: human epididymis protein 4
WFDC2: WAP four-disulfide core protein 2
SLPI: secretory leucocyte protease inhibitor
ANXA2/A2: annexin A2
CA125: carbohydrate antigen 125
MMP2: matrix metalloproteinase 2
MMP9: matrix metalloproteinase 9
EGFR: epidermal growth factor receptor
IGF1R: insulin-like growth factor 1 receptor
HIF1α: hypoxia inducible factor 1α
EGF: epidermal growth factor
VEGF: vascular endothelial growth factor
MAPK: mitogen-activated protein kinase
PI3K: phosphoinositide 3-kinase
AKT: protein kinase B, PKB
ERK1/2: extracellular signal-regulated kinase 1/2

## Availability of data and materials

The datasets used or analysed during the current study are available from the corresponding author upon reasonable request.
